# A study on the impact of childhood obesity on health-related physical fitness through motor coordination–related functional pathways

**DOI:** 10.3389/fnut.2026.1776788

**Published:** 2026-01-29

**Authors:** Deqiang Zhao, Xiaoxiao Chen, Xiang Pan, Shuwan Wang, Jiameng Wang, Haixia Hu, Yanfeng Zhang

**Affiliations:** 1 China Institute of Sport Science, Beijing, China; 2 School of Physical Education and Sports Rehabilitation, Jinzhou Medical University, Jinzhou, Liaoning, China; 3 Graduate School of Health and Sports Science, Juntendo University, Inzai, Japan; 4 School of Exercise and Health, Shanghai University of Sport, Shanghai, China; 5 Aiyoudong Children and Youth Sports Health Research Institute, Weifang, Shandong, China

**Keywords:** body fat percentage, childhood obesity, health-related physical fitness, mediating effect, motor coordination

## Abstract

**Objective:**

This study aimed to investigate the impact of childhood obesity on health-related physical fitness performance and to examine the mediating role of motor coordination ability as a behavioral functional pathway in this relationship.

**Methods:**

A cross-sectional study design was employed. In June 2025, 431 children aged 7–14 years (204 in the obesity group, 227 in the normal-weight group) were recruited from Weifang City, Shandong Province, China. Body composition (BMI and PBF) was measured using a bioelectrical impedance analyzer. Health-related physical fitness index was assessed according to the Chinese National Student Physical Fitness Standard and synthesized into a standardized physical fitness index. Motorcoordination was evaluated using the Movement Assessment Battery for Children-2 (MABC-2), which reflects behavioral motor performance. Statistical analyses included independent samples *t*-tests, Pearson correlation analysis, hierarchical regression analysis, and the Bootstrap method for mediation effect testing.

**Results:**

Both the physical fitness index and the motor coordination index were significantly lower in obese children compared to normal-weight children (Cohen’s *d* = 0.31–0.34). BMI and PBF showed significant negative correlations with the physical fitness index. After controlling for area (urban/rural), gender, and age, both BMI and PBF independently and negatively predicted the physical fitness index. Mediation analysis indicated that motor coordination played a statistically significant partial mediating role in the relationship between BMI and the physical fitness index (indirect effect = −0.060, 95% CI [−0.105, −0.048]), as well as between PBF and the physical fitness index (indirect effect = −0.036, 95% CI [−0.052, −0.022]).

**Conclusion:**

Childhood obesity is closely associated with decreased health-related physical fitness, with PBF being a more stable predictor than BMI. Motor coordination represents one behavioral pathway that may partially explain the association between body composition and physical fitness performance. Given the cross-sectional design, these findings reflect statistical associations rather than causal or neuromotor mechanisms. Longitudinal and intervention studies are needed to further verify directionality.

## 1 Introduction

Childhood obesity is widely recognized as one of the most pressing global public health challenges, with its prevalence rising continuously among children and adolescents (1–3). Extensive epidemiological evidence indicates that childhood obesity not only significantly elevates the risk of chronic diseases such as cardiovascular disease and type 2 diabetes in adulthood, but also exerts broad and profound negative effects on the musculoskeletal system, cardiopulmonary function, and motor competence during crucial growth and developmental periods (4–7). These adverse outcomes are frequently associated with reduced motor ability and deteriorated physical fitness in children, which may further diminish their engagement in physical activity, potentially establishing a detrimental cycle (8).

Against this backdrop, children’s health-related physical fitness has garnered considerable attention (9). It is not only a core indicator of physical health but also an important predictor of future cardiovascular metabolic risk, psychological health, and even academic performance (10, 11). Concurrently, motor competence, as the foundation for children’s participation in sports activities and mastery of motor skills, is also closely related to weight status (12, 13). Traditional perspectives have predominantly focused on the impact of obesity on basic fitness components such as strength and endurance. However, recent research has begun to reveal that obesity may similarly be associated with lower performance in more complex motor functions such as fine motor control, dynamic balance, and hand-eye coordination (14). Studies have shown non-linear relationships between BMI and motor coordination in children (15), and benefits of normal BMI on physical fitness (16). Research in preschool and young school-age children also indicates relationships between body composition, physical activity, and fundamental motor skills (17–20).

Nevertheless, existing research has several limitations. Firstly, most studies use body mass index (BMI) as the sole indicator of obesity (20). While BMI is convenient for screening, it cannot distinguish between muscle mass and fat mass, potentially masking the heterogeneous effects of different body compositions on motor ability. Body fat percentage (PBF), as a more direct indicator of fat load, requires more detailed analysis regarding its relationship with physical fitness performance. Secondly, when exploring the relationship between obesity and physical fitness, research has mostly focused on direct associations, with less exploration of potential intermediary factors (21, 22). Motor competence, especially the fine motor and coordination skills requiring complex neuromuscular integration, may be a key factor connecting body composition to overall physical fitness performance (23, 24). Obesity may be linked to children’s performance in various fitness tests through its association with movement coordination and efficiency (25). Finally, large-sample empirical studies focusing on children and adolescents, particularly those incorporating BMI, PBF, motor coordination, and comprehensive physical fitness assessments, remain relatively limited.

Existing research generally acknowledges the adverse associations of obesity with children’s motor performance and physical fitness levels, observing consistent negative correlations across different fitness dimensions (26, 27). However, such studies often focus on motor outcomes, emphasizing the descriptive association, (28), while less frequently delving into the underlying statistical pathways. Obesity may be linked to children’s physical fitness performance through factors such as altered body structure characteristics and movement execution efficiency, but these pathways lack systematic empirical examination (29, 30). Particularly regarding the integration of body composition characteristics and motor coordination ability into the same analytical model to explore the statistical pathway, related research is still insufficient.

**Figure 1:**
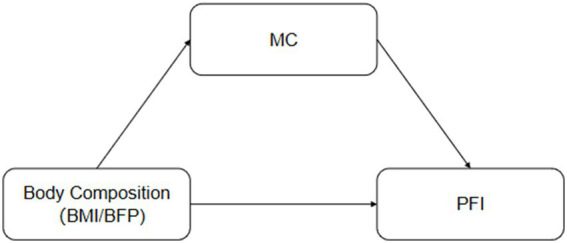
Hypothetical path: Body composition, motor coordination, and physical fitness index.

Based on this, the present study targets Chinese children aged 7–14 years and is structured around a conceptual framework (see Figure 1) in which body composition (assessed via BMI and PBF) is hypothesized to influence health-related physical fitness index (PFI) both directly and indirectly through motor coordination ability (MC). Specifically, the study will (1) systematically compare differences in PFI and MC between obese and normal-weight children; (2) separately examine the independent predictive roles of BMI and PBF on PFI; and (3) test whether MC mediates the relationship between body composition and PFI. By examining these pathways, this study aims to elucidate the potential mechanisms linking childhood obesity to poorer physical fitness from a motor coordination perspective, thereby offering more targeted scientific evidence for designing integrated intervention strategies that combine weight management with motor skill development.

## 2 Methods

### 2.1 Participants

This study employed a cross-sectional design and was conducted in June 2025 across 4 primary schools and 2 middle schools in Weifang City, Shandong Province, China. The participants were school children aged 7–14 years. To systematically compare differences between obese and normal-weight children and meet statistical requirements, a targeted cluster recruitment strategy was adopted, aiming to enroll children into an obesity group and a normal-weight group at an approximate 1:1 ratio, with a target sample size of 460. The specific recruitment procedure was as follows: First, based on school physical examination and fitness test data, children meeting the World Health Organization criteria for childhood obesity (obesity group) and those within the normal weight range (normal-weight group) were preliminarily identified. Subsequently, study information and informed consent forms were distributed to the guardians of potentially eligible children through the schools. Inclusion criteria were: (1) age 7–14 years; (2) the ability to participate normally in physical activities. Exclusion criteria were: (1) a diagnosed central nervous system diseases; (2) severe cardiovascular or pulmonary diseases; (3) a musculoskeletal disorders or injuries affecting motor performance; (4) a intellectual or developmental disabilities. Finally, informed consent was obtained from the guardians of all participating children. This study was approved by the Ethics Committee of the China Institute of Sport Science, General Administration of Sport of China (Approval No.: CISSLA20250530).

After excluding cases with missing key indicators, a total of 431 children completed all tests and were included in the statistical analysis, comprising 204 in the obesity group and 227 in the normal weight group. Based on a medium effect size (*f*^2^ = 0.05) for the obesity-physical fitness relationship from previous literature, sample size estimation was performed using G*Power 3.1 software (31). With a significance level *α* = 0.05, statistical power (1−*β*) = 0.80, and 6 predictor variables, the minimum required sample size was approximately 280. The actual sample size of 431 in this study meets the requirements for statistical power in mediation models and multiple regression analyses.

### 2.2 Measures and instruments

#### 2.2.1 Body composition measurement

Height and weight were measured using a multi-frequency bioelectrical impedance analyzer (InBody 770), from which body mass index (BMI, kg/m^2^) and body fat percentage (PBF, %) were directly calculated. This device has good reliability and validity and is widely used for body composition assessment in children (32).

#### 2.2.2 Health-related physical fitness index (PFI)

Testing covered all students from grades 1 to 9 (primary school grade 1 to junior high school grade 3). According to the grade-grouping regulations of the Chinese National Student Physical Fitness Standard (33), specific test items were as follows: Grades 1–2: 50-m run, sit-and-reach, 1-min rope skipping. Grades 3–4: 50-m run, sit-and-reach, 1-min rope skipping, 1-min sit-ups. Grades 5–6: 50-m run, sit-and-reach, 1-min rope skipping, 1-min sit-ups, 50 m × 8 shuttle run. Grades 7–9: 50-m run, sit-and-reach, standing long jump, pull-ups (boys)/1-min sit-ups (girls), 1,000-m run (boys)/800-m run (girls). Final scores were standardized into Z-scores specific to age and gender and then summed to represent children’s health-related physical fitness index.

#### 2.2.3 Motor coordination (MC)

Standardized testing was conducted using the Movement Assessment Battery for Children-2 (MABC-2). This tool sets tasks according to age bands, assessing three dimensions: manual dexterity, static and dynamic balance, and aiming & catching (hand-eye coordination and object control). Raw scores for each item were converted into age-specific Z-scores, and the Z-scores from all dimensions were summed to obtain a comprehensive motor coordination index (34).

### 2.3 Testing procedure

All tests were completed within 14 days. Body composition measurement, health-related physical fitness assessment, and motor coordination testing were all conducted by a team of physical education teachers who underwent unified, rigorous professional training. All procedures strictly followed the standardized operation manuals of the respective assessment tools. Testing took place simultaneously in the gymnasiums or standard playgrounds of the students’ respective schools to ensure standardized environments and equipment. Logistically, body composition and motor coordination tests were conducted in the morning, followed by health-related physical fitness tests in the afternoon.

### 2.4 Statistical analysis

All analyses were performed using SPSS 27.0 and the PROCESS v3.5 macro. Firstly, descriptive statistics (mean, standard deviation) were calculated for each group. Secondly, independent samples *t*-tests were used to compare differences in PFI and MC between the obesity and normal weight groups, and Cohen’s d was calculated to assess effect sizes. Next, Pearson correlation analysis was used to explore the relationships among BMI, PBF, MC, and PFI. Subsequently, hierarchical multiple linear regression analysis was conducted: after controlling for urban/rural location, gender, and age, BMI and PBF were separately entered into the models to examine their independent predictive effects on PFI. Finally, the bias-corrected percentile Bootstrap method (5,000 resamples) was used to test the mediating effect of MC in the relationship between BMI/PBF and PFI, reporting the point estimate of the indirect effect and its 95% confidence interval.

## 3 Results

### 3.1 Sample characteristics

This study included a total of 431 children, comprising 204 in the obesity group and 227 in the normal weight group. Sample characteristics are detailed in Table 1.

**Table 1:** Descriptive statistical analysis of the sample.

Weight status	Variable	*N*	Min	Max	Mean	SD
Normal	Age	227	7	14	10.53	1.99
	Height (cm)	227	121	180	146.17	14.16
	Weight (kg)	227	18.85	68	36.09	10.57
	BMI (kg/m^2^)	227	13.3	30.3	17.97	3.23
	PBF	227	3	77.2	20.27	9.98
	PFI	227	−12.46	10.37	0.11	2.72
	MC	227	−49.5	50.5	−1.64	12.07
	Urban/rural	227	1	2	1.69	0.49
Obese	Age	204	7	14	10.62	2.18
	Height (cm)	204	123	182	151.14	14.86
	Weight (kg)	204	21.18	137.24	56.13	19.21
	BMI (kg/m^2^)	204	14.1	33.3	24.13	5.03
	PBF	204	9.5	50.2	31.41	9.33
	PFI	204	−5.93	7.5	−0.13	2.35
	MC	204	−49	19.5	−5.59	11.53
	Urban/rural	204	1	2	1.50	0.50

### 3.2 Correlations among variables

The correlation matrix (Table 2) indicated that BMI and PBF were strongly positively correlated (*r* = 0.750, *p* < 0.01). BMI was significantly negatively correlated with MC (*r* = −0.263, *p* < 0.01). PBF was significantly negatively correlated with PFI (*r* = −0.135, *p* < 0.05). PBF was significantly negatively correlated with MC (*r* = −0.177, *p* < 0.01). MC was significantly positively correlated with PFI (*r* = 0.119, *p* < 0.05).

**Table 2:** Correlation matrix for BMI, body fat percentage (PBF), motor coordination (MC), and physical fitness (PFI).

Variable	BMI	PBF	MC	PFI
BMI	1			
PBF	0.750^**^	1		
MC	−0.263^**^	−0.177^**^	1	
PFI	−0.186^**^	−0.135^*^	0.119^*^	1

^*^p < 0.05, ^**^p < 0.01.

### 3.3 Comparison of MC and PFI between normal weight and obese children

Independent samples *t*-test results (Table 3) showed that the comprehensive physical fitness index (PFI) of obese children was significantly lower than that of normal-weight children (*t* = 3.372, *p* = 0.001, Cohen’s *d* = 0.315). Simultaneously, the comprehensive motor coordination index (MC) of obese children was also significantly lower (*t* = 3.469, *p* = 0.001, Cohen’s *d* = 0.335).

**Table 3:** Independent samples *t*-test comparing physical fitness (PFI) and motor coordination (MC) between normal weight and obese children.

Variable	Group	*N*	Mean (±SD)	*t*	*p*	Cohen’s *d*	95% CI
PFI	Normal	227	0.11 (±2.72)	3.372	0.001	0.315	[0.194, 0.485]
PFI	Obese	204	−0.13 (±2.35)				
MC	Normal	227	−1.64 (±12.07)	3.469	0.001	0.335	[0.144, 0.525]
MC	Obese	204	−5.59 (±11.53)				

### 3.4 Regression analysis results

Hierarchical regression analysis (Table 4) showed that after controlling for urban/rural location, gender, age, and MC, BMI had a significant negative predictive effect on PFI (*β* = −0.116, *p* = 0.032). Similarly, PBF also had an independent negative predictive effect on PFI (*β* = −0.114, *p* = 0.026). Notably, MC was a significant positive predictor of PFI in both models (*p* < 0.01).

**Table 4:** Regression analysis results for physical fitness (PFI).

Model	Variable	SE	*β*	*t*	*p*	*B*	95%CI lower	95%CI upper
BMI	Urban/rural	0.185	0.092	0.102	2.011	0.045	0.004	0.366
	Sex	0.112	0.274	0.021	0.41	0.682	−0.427	0.651
	Age	0.211	0.067	0.173	3.134	0.002	0.078	0.343
	MC	0.034	0.012	0.161	2.893	0.004	0.011	0.057
	BMI	−0.057	0.026	−0.116	−2.151	0.032	−0.108	−0.005
	*R*^2^ = 0.347, Δ*R*^2^ = 0.337, *F* = 4.679, *p* = 0.001							
PBF	Urban/rural	0.178	0.09	0.099	1.978	0.048	0.001	0.355
	Sex	0.022	0.269	0.004	0.08	0.936	−0.508	0.551
	Age	0.173	0.067	0.143	2.605	0.01	0.043	0.304
	MC	0.036	0.012	0.168	3.05	0.002	0.013	0.059
	PBF	−0.026	0.012	−0.114	−2.242	0.026	−0.049	−0.003
	*R*^2^ = 0.348, Δ*R*^2^ = 0.338, *F* = 4.783, *p* = 0.001							

### 3.5 Mediation effect analysis

**Figure 2:**
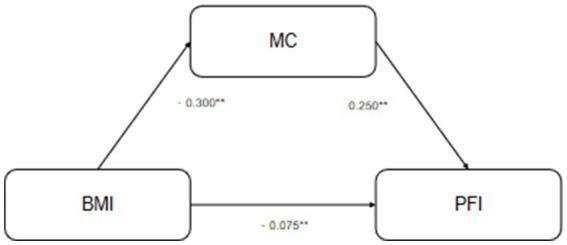
Mediation pathway of BMI’s effect on PFI.

**Figure 3:**
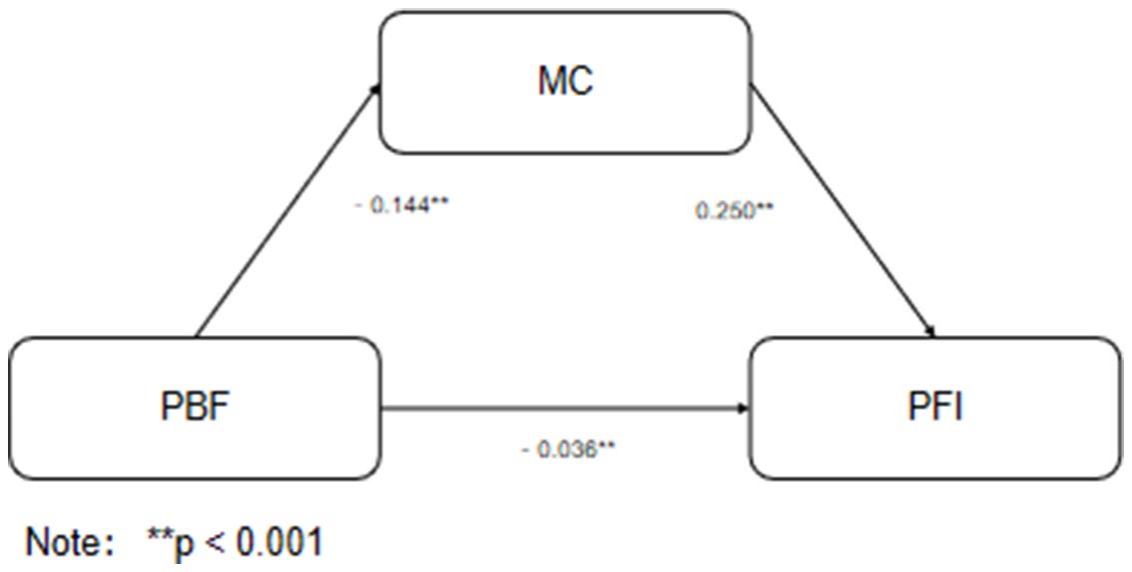
Mediation pathway of PBF’s effect on PFI.

The mediating role of motor coordination ability (MC) in the relationship between body composition indicators (BMI and body fat percentage, PBF) and health-related physical fitness (PFI) was examined using the bias-corrected percentile Bootstrap method (with 5,000 resamples). Detailed results are presented in Figures 2, 3, and Table 5.

**Table 5:** Mediation effect analysis of motor coordination (MC) in the relationship between BMI/body fat percentage and physical fitness index (PFI).

Path	Effect type	Effect estimate	SE	95% CI	*p*/Conclusion
BMI → PFI	Total effect	−0.150	0.025	[−0.199, −0.101]	0.001^**^
BMI → PFI	Direct effect	−0.075	0.022	[−0.118, −0.032]	0.001^**^
BMI → MC → PFI	Indirect effect	−0.060	0.015	[−0.105, −0.048]	Partial mediation
PBF → PFI	Total effect	−0.120	0.018	[−0.155, −0.085]	0.006^**^
PBF → PFI	Direct effect	−0.084	0.017	[−0.117, −0.051]	0.009^**^
PBF → MC → PFI	Indirect effect	−0.036	0.008	[−0.052, −0.022]	Partial mediation

For BMI, its total effect on PFI was significant (*β* = −0.145, *p* = 0.001). The direct effect of BMI on PFI remained significant (*β* = −0.075, *p* = 0.001), and the indirect effect through MC was also significant (*β* = −0.060, 95% CI [−0.105, −0.048]). This indicates that MC plays a partial mediating role in the relationship between BMI and PFI.

Similarly, for body fat percentage (PBF), its total effect on PFI was significant (*β* = −0.120, *p* = 0.006). The direct effect of PBF on PFI was significant (*β* = −0.084, *p* = 0.009), and the indirect effect through MC was also significant (*β* = −0.036, 95% CI [−0.052, −0.022]), indicating that MC plays a partial mediating role.

In both models, motor coordination ability served as a significant partial mediator, partly accounting for the negative association between body composition (BMI and PBF) and physical fitness in children within this cross-sectional data.

## 4 Discussion

Based on a large sample of Chinese children, this study systematically examined the relationships between body composition, motor coordination, and health-related physical fitness, and tested a statistical pathway linking childhood obesity to fitness performance. The results confirm that obese children tend to have lower physical fitness and motor coordination. Importantly, this study makes a key methodological contribution by integrating both PBF and motor coordination within a single analytical model, thereby offering a more refined understanding of the functional pathways connecting obesity and fitness.

### 4.1 The relationship between childhood obesity and decline in health-related physical fitness

The results indicate that obese children have a significantly lower comprehensive physical fitness index than their normal-weight peers, with a small-to-medium effect size. After controlling for age, gender, and urban/rural factors, both BMI and PBF remained independently and negatively associated with physical fitness, though the associations were modest in size. This suggests that body composition is one of several factors influencing fitness. These findings align with previous evidence that obesity is linked to poorer fitness outcomes in childhood (35, 36). Studies have similarly shown benefits of normal BMI on physical fitness in children (16).

From a biomechanical perspective, excess adipose tissue may increase mechanical load during dynamic tasks, reducing movement efficiency (37). In fitness tests involving rapid displacement and repetitive load-bearing, higher fat mass may elevate movement difficulty (38). Obese children may also be more prone to fatigue accumulation, limiting performance (39). Notably, PBF demonstrated a more stable association with physical fitness than BMI, suggesting that fat mass itself is an important factor related to children’s physical fitness. This supports the use of body fat-related indicators in childhood obesity research.

### 4.2 Childhood obesity and impaired motor coordination ability

This study found that the comprehensive motor coordination index of obese children was significantly lower than that of normal-weight children (Cohen’s *d* = 0.335), and BMI was significantly negatively correlated with motor coordination ability (*r* = −0.263). This result is consistent with literature indicating that obesity is associated with poorer motor performance in children (40), including non-linear relationships between BMI and motor coordination (15). From a developmental perspective, childhood is a critical stage for the development of motor control and coordination abilities (40). Excess fat may be related to challenges in this process: on one hand, fat accumulation may alter the body’s center of gravity, affecting balance ability (41); on the other hand, obesity may be associated with reduced precision of movement execution (42). Furthermore, obese children may show differences in brain regions associated with movement, offering a neurobiological perspective for the link between obesity and motor coordination (43–45).

Motor coordination ability is not only an outcome of motor skill development but also a crucial factor for children’s participation in physical activities. Children with lower motor coordination are more likely to experience difficulties in physical activities, potentially reducing their motivation to engage in sports and further decreasing physical activity participation.

### 4.3 The statistical mediating role of motor coordination ability

An important finding is that motor coordination ability plays a partial mediating role in the statistical association between body composition (both BMI and PBF) and health-related physical fitness. The indirect effects were small (*β* = −0.060 for BMI and *β* = −0.036 for PBF). This suggests a potential functional pathway that may help explain why obese children tend to perform poorer in fitness tests, beyond the simple effect of increased weight. From a motor control perspective, fitness tests rely not only on muscular strength and cardiorespiratory endurance but also depend on movement coordination and stability (46). For example, in the 50-m run and shuttle run, stride regulation, trunk stability, and coordination may impact performance. Due to lower motor coordination ability, obese children may struggle to perform these movements as efficiently, placing them at a disadvantage. Similar mediating or correlational roles of motor competence have been observed in other studies examining fitness and weight status in children (16, 40, 47, 48).

Although the mediation effect was relatively small, its stable existence suggests that motor coordination is one potential pathway through which body composition is associated with physical fitness (49, 50). This highlights the multifactorial nature of fitness development. The effect sizes, while statistically significant, are modest, and their practical impact should be considered alongside statistical significance.

## 5 Strengths and limitations

This study has several strengths: (1) the simultaneous use of both BMI and PBF to characterize body composition multidimensionally; (2) the introduction of motor coordination as a mediator to systematically examine the pathway from body composition to physical fitness; and (3) a relatively large sample spanning multiple age groups, with assessments conducted according to national standards.

Several limitations should also be noted. First, the cross-sectional design precludes causal inference; the mediation analysis reveals statistical associations rather than established causal mechanisms. Longitudinal or intervention studies are needed to verify temporal and causal pathways. Second, potential confounders—such as physical activity, sedentary behavior, pubertal status, and socioeconomic details—were not included. Thus, motor coordination represents one possible explanatory factor, not the exclusive mechanism linking obesity to fitness. Future studies should incorporate these multidimensional indicators to build more comprehensive models. Third, motor coordination was assessed behaviorally (MABC-2); direct neuromotor or neurophysiological measures (e.g., EMG, neuroimaging) were not collected.

## 6 Conclusion

This study indicates that childhood obesity is closely associated with decreased health-related physical fitness, with both BMI and body fat percentage showing independent negative associations. Motor coordination ability plays a partial mediating role in the statistical association between body composition and physical fitness, suggesting it may be one functional pathway involved. This study systematically examines the “body composition → motor coordination → physical fitness” pathway, providing a perspective for childhood obesity intervention that emphasizes the potential value of integrating motor skill training with weight management approaches.

## Data Availability

The raw data supporting the conclusions of this article will be made available by the authors, without undue reservation.

## References

[1] Dong Y Yuan C Dang J Song X Cheng G Chen Y . Control of childhood obesity and implications for policy in China. Lancet Public Health. (2024) 9:e1125–35. doi: 10.1016/S2468-2667(24)00263-9, 39579776

[2] Karnik S Kanekar A. Childhood obesity: a global public health crisis. Int J Prev Med. (2012) 3:1–7. 22506094 PMC3278864

[3] Ou-Yang X Zhang G Li J Zou N Yuan L Yi H. Weighing the future: strategic measures against rising childhood obesity. Pediatr Res. (2025) 97:1795–7. doi: 10.1038/s41390-024-03626-1, 39379625

[4] Salama M Balagopal B Fennoy I Kumar S. Childhood obesity, diabetes. And cardiovascular disease risk. J Clin Endocrinol Metab. (2023) 108:3051–66. doi: 10.1210/clinem/dgad361, 37319430

[5] Hertiš Petek T Marčun Varda N. Childhood cardiovascular health, obesity, and some related disorders: insights into chronic inflammation and oxidative stress. Int J Mol Sci. (2024) 25:9706. doi: 10.3390/ijms25179706, 39273654 PMC11396019

[6] Chung ST Krenek A Magge SN. Childhood obesity and cardiovascular disease risk. Curr Atheroscler Rep. (2023) 25:405–15. doi: 10.1007/s11883-023-01111-4, 37256483 PMC10230147

[7] Orhan BE Karaçam A AlKasasbeh WJ Amawi AT Canli U. Parental feeding perceptions and family health behaviours: an analysis based on nutrition and physical activity tendencies. Front Pediatr. (2026) 13:1704116. doi: 10.3389/fped.2025.1704116 41624819 PMC12855539

[8] Şendil AM Canlı U Sheeha BB Alkhamees NH Batrakoulis A Al-Mhanna SB. The effects of structured coordinative exercise protocol on physical fitness, motor competence and inhibitory control in preschool children. Sci Rep. (2024) 14:28462. doi: 10.1038/s41598-024-79811-3, 39558052 PMC11574278

[9] Wang W Huang Z Gong J Li Q Liu G Ge R. Physical fitness status and associated determinants among Chinese children aged 9–12 years in Shandong province: a population-based cross-sectional study. Sci Rep. (2025) 15:29221. doi: 10.1038/s41598-025-13319-2, 40783637 PMC12335582

[10] Silva DAS Tremblay MS. Health-related physical fitness in children and adolescents and the United Nations sustainable development goals: signals of convergence. J Sport Health Sci. (2025) 15:101105. doi: 10.1016/j.jshs.2025.101105, 41314463 PMC12830232

[11] Sember V Jurak G Kovač M Morrison SA Starc G. Children's physical activity, academic performance, and cognitive functioning: a systematic review and meta-analysis. Front Public Health. (2020) 8:307. doi: 10.3389/fpubh.2020.00307 32760689 PMC7372103

[12] Sepúlveda C Monsalves-Álvarez M Troncoso R Weisstaub G. Children and adolescents with overweight or obesity exhibit poor cardiorespiratory performance and elevated energy expenditure during an exercise task. PLoS One. (2025) 20:e0327875. doi: 10.1371/journal.pone.0327875, 40627658 PMC12237028

[13] Morrison KM Cairney J Eisenmann J Pfeiffer K Gould D. Associations of body mass index, motor performance, and perceived athletic competence with physical activity in normal weight and overweight children. J Obes. (2018) 2018:3598321. doi: 10.1155/2018/3598321, 29854437 PMC5954868

[14] Wang C Chan JS Ren L Yan JH. Obesity reduces cognitive and motor functions across the lifespan. Neural Plast. (2016) 2016:2473081. doi: 10.1155/2016/2473081, 26881095 PMC4737453

[15] Lopes VP Malina RM Maia JAR Rodrigues LP. Body mass index and motor coordination: non-linear relationships in children 6-10 years. Child Care Health Dev. (2018) 44:443–51. doi: 10.1111/cch.12557, 29417602

[16] Bi C Yang J Sun J Song Y Wu X Zhang F. Benefits of normal body mass index on physical fitness: a cross-sectional study among children and adolescents in Xinjiang Uyghur autonomous region, China. PLoS One. (2019) 14:e0220863. doi: 10.1371/journal.pone.0220863, 31415603 PMC6695220

[17] Ma FF Luo DM. Relationships between physical activity, fundamental motor skills, and body mass index in preschool children. Front Public Health. (2023) 11:1094168. doi: 10.3389/fpubh.2023.1094168, 37124831 PMC10130375

[18] Marmeleira J Veiga G Cansado H Raimundo A. Relationship between motor proficiency and body composition in 6- to 10-year-old children. J Paediatr Child Health. (2017) 53:348–53. doi: 10.1111/jpc.13446, 28045215

[19] Matarma T Lagström H Hurme S Tammelin TH Kulmala J Barnett LM . Motor skills in association with physical activity, sedentary time, body fat, and day care attendance in 5-6-year-old children-the STEPS study. Scand J Med Sci Sports. (2018) 28:2668–76. doi: 10.1111/sms.13264, 30003602

[20] Ayuzo Del Valle NC Pérez-Treviño P Cepeda Lopez AC Murillo-Torres RM Castillo EC Gutierrez-Cantu D . Beyond BMI: a comprehensive approach to pediatric obesity assessment. Front Pediatr. (2025) 13:1597309. doi: 10.3389/fped.2025.1597309, 40487022 PMC12142046

[21] Barros WMA Silva KG Silva RKP Souza APS Silva ABJ Silva MRM . Effects of overweight/obesity on motor performance in children: a systematic review. Front Endocrinol. (2022) 12:759165. doi: 10.3389/fendo.2021.759165, 35126307 PMC8812008

[22] Martins JMC Landeiro JC Cardoso JN Honório SAA. The influence of obesity on the motor coordination in children between 6 and 9 years of age. Sci Sports. (2022) 37:564–71. doi: 10.1016/j.scispo.2022.01.006

[23] Zhou Y Tolmie A. Associations between gross and fine motor skills, physical activity, executive function, and academic achievement: longitudinal findings from the UK millennium cohort study. Brain Sci. (2024) 14:121. doi: 10.3390/brainsci14020121, 38391696 PMC10887312

[24] Bondi D Robazza C Lange-Küttner C Pietrangelo T. Fine motor skills and motor control networking in developmental age. Am J Hum Biol. (2022) 34:e23758. doi: 10.1002/ajhb.23758, 35613316 PMC9541226

[25] Ge S Liu H Song C Zhang W Guo X. Evaluating the impact of motor quotient physical fitness training on health-related fitness indicators and obesity risk in children aged 7–8 years in Tianjin, China. BMC Public Health. (2025) 25:739. doi: 10.1186/s12889-025-21985-0, 39987081 PMC11847385

[26] Aniśko B Bernatowicz K Wójcik M. Effects of body mass index and extracurricular sports activities on physical fitness in school-aged children. Front Public Health. (2025) 13:1578304. doi: 10.3389/fpubh.2025.1578304 40352849 PMC12061684

[27] Rauner A Mess F Woll A. The relationship between physical activity, physical fitness and overweight in adolescents: a systematic review of studies published in or after 2000. BMC Pediatr. (2013) 13:19. doi: 10.1186/1471-2431-13-19, 23375072 PMC3571910

[28] Qin C Fan C Wang J Li Q Liu J Wang H . Trends and inequalities in physical fitness and nutritional status among 0.72 million Chinese adults aged 20–59 years: an analysis of five successive national surveillance surveys, 2000–2020. Lancet Region Health Western Pac. (2025) 57:101542. doi: 10.1016/j.lanwpc.2025.101542, 40242466 PMC12000743

[29] Guo Y Jiang S Li H Xie G Pavel V Zhang Q . Obesity induces osteoimmunology imbalance: molecular mechanisms and clinical implications. Biomed Pharmacother. (2024) 177:117139. doi: 10.1016/j.biopha.2024.117139, 39018871

[30] Zhang D Shen C Chen N Liu C Hu J Lau KK . Long-term obesity impacts brain morphology, functional connectivity and cognition in adults. Nat Ment Health. (2025) 3:466–78. doi: 10.1038/s44220-025-00396-5

[31] Ding C Jiang Y. The relationship between body mass index and physical fitness among Chinese university students: results of a longitudinal study. Healthcare (Basel). (2020) 8:570. doi: 10.3390/healthcare8040570, 33348642 PMC7765873

[32] Looney DP Schafer EA Chapman CL Pryor RR Potter AW Roberts BM . Reliability, biological variability, and accuracy of multi-frequency bioelectrical impedance analysis for measuring body composition components [original research]. Front Nutr. (2024) 11:1491931. doi: 10.3389/fnut.2024.1491931 39691170 PMC11649400

[33] Zhang F Bi C Yin X Chen Q Li Y Liu Y . Physical fitness reference standards for Chinese children and adolescents. Sci Rep. (2021) 11:4991. doi: 10.1038/s41598-021-84634-7, 33654251 PMC7925655

[34] Hua L Song Z Liu J Zhang B. Intervention effect of physical activity on motor coordination in children and adolescents with developmental coordination disorder: a meta-analysis. Front Psychol. (2025) 16:1630439. doi: 10.3389/fpsyg.2025.1630439 41346498 PMC12672234

[35] Podogrodzki J Szalecki M Wrona A Wierzbicka-Rucińska A. Assessment of physical fitness in children and adolescents with simple obesity. Children (Basel). (2025) 12:1388. doi: 10.3390/children12101388, 41153570 PMC12563853

[36] Morano M Robazza C Bortoli L Rutigliano I Ruiz MC Campanozzi A. Physical activity and physical competence in overweight and obese children: an intervention study. Int J Environ Res Public Health. (2020) 17:17176370. doi: 10.3390/ijerph17176370, 32883044 PMC7504542

[37] Menendez A Wanczyk H Walker J Zhou B Santos M Finck C. Obesity and adipose tissue dysfunction: from pediatrics to adults. Genes (Basel). (2022) 13:1866. doi: 10.3390/genes13101866, 36292751 PMC9601855

[38] Bowser BJ Roles K. Effects of overweight and obesity on running mechanics in children. Med Sci Sports Exerc. (2021) 53:2101–10. doi: 10.1249/mss.0000000000002686, 33867501

[39] Singh B Takeda MM Niino MF Goulart JD Hammons AJ Roos JM . The effects of adiposity, muscular strength, cardiorespiratory fitness, and fatigue on gait biomechanics in overweight and obese children. Clin Biomech. (2021) 84:105332. doi: 10.1016/j.clinbiomech.2021.105332, 33819825

[40] Zacks B Confroy K Frino S Skelton JA. Delayed motor skills associated with pediatric obesity. Obes Res Clin Pract. (2021) 15:1–9. doi: 10.1016/j.orcp.2020.10.003, 33268277

[41] Son SM. Influence of obesity on postural stability in young adults. Osong Public Health Res Perspect. (2016) 7:378–81. doi: 10.1016/j.phrp.2016.10.001, 28053843 PMC5194219

[42] Teasdale N Simoneau M Corbeil P Handrigan G Tremblay A Hue O. Obesity alters balance and movement control. Curr Obes Rep. (2013) 2:235–40. doi: 10.1007/s13679-013-0057-8

[43] Mora-Gonzalez J Esteban-Cornejo I Cadenas-Sanchez C Migueles JH Molina-Garcia P Rodriguez-Ayllon M . Physical fitness, physical activity, and the executive function in children with overweight and obesity. J Pediatr. (2019) 208:50–56.e1. doi: 10.1016/j.jpeds.2018.12.028, 30902422

[44] Morys F Tremblay C Rahayel S Hansen JY Dai A Misic B . Neural correlates of obesity across the lifespan. Commun Biol. (2024) 7:656. doi: 10.1038/s42003-024-06361-9, 38806652 PMC11133431

[45] Zhao S Semeia L Veit R Moser J Preissl H Kullmann S. Structural and functional brain changes in children and adolescents with obesity. Obes Rev. (2025) 26:e70001. doi: 10.1111/obr.70001 40759532 PMC12620109

[46] Hirono T Okudaira M Takeda R Ueda S Nishikawa T Igawa K . Association between physical fitness tests and neuromuscular properties. Eur J Appl Physiol. (2024) 124:1703–17. doi: 10.1007/s00421-023-05394-y, 38193907

[47] Cheng J East P Blanco E Sim EK Castillo M Lozoff B . Obesity leads to declines in motor skills across childhood. Child Care Health Dev. (2016) 42:343–50. doi: 10.1111/cch.12336, 27059409 PMC4841726

[48] Ružbarská I. Gross motor coordination and physical fitness in overweight and obese primary school children compared with normal weight peers. Phys Act Rev. (2024) 12: 95–101. doi: 10.16926/par.2024.12.21

[49] C D’A Forte P Pugliese E. Trends in physical activity and motor development in young people—decline or improvement? A review. Children. (2024) 11:298. doi: 10.3390/children11030298 38539333 PMC10969615

[50] D'Hondt E Deforche B Gentier I Verstuyf J Vaeyens R De Bourdeaudhuij I . A longitudinal study of gross motor coordination and weight status in children. Obesity. (2014) 22:1505–11. doi: 10.1002/oby.20723, 24549983

